# The Evolution of Diabetes Treatment Through the Ages: From Starvation Diets to Insulin, Incretins, SGLT2-Inhibitors and Beyond

**DOI:** 10.1007/s41745-023-00357-w

**Published:** 2023-02-21

**Authors:** Sunder Mudaliar

**Affiliations:** 1 Veterans Affairs Medical Center, San Diego, CA USA; 2 Diabetes/Metabolism Section, VA San Diego HealthCare System, Department of Medicine, San Diego School of Medicine, University of California at San Diego, 3350 La Jolla Village Drive (Mail Code: 111G), San Diego, CA 92161 USA

## Abstract

Diabetes is an ancient disease and for centuries extreme diets and herbal remedies were used to treat diabetes symptoms. The discovery of insulin in 1921 transformed the landscape of diabetes treatment and was followed by the discovery of several new therapies which improved glycemia and increased patient life span. However, as patients with diabetes lived longer, they developed classic microvascular and macrovascular diabetes complications. In the 1990s, the DCCT and the UKPDS trials demonstrated that tight glucose control reduced the microvascular complications of diabetes, but had marginal effects on cardiovascular disease, the leading cause of death in patients with diabetes. In 2008, the FDA directed that all new diabetes medications demonstrate cardiovascular safety. From this recommendation emerged novel therapeutic classes, the GLP-1 receptor agonists and SGLT2-Inhibitors, which not only improve glycemia, but also provide robust cardio-renal protection. In parallel, developments in diabetes technology like continuous glucose monitoring systems, insulin pumps, telemedicine and precision medicine have advanced diabetes management. Remarkably, a century later, insulin remains a cornerstone of diabetes treatment. Also, diet and physical activity remain important components of any diabetes treatment. Today type 2 diabetes is preventable and long-term remission of diabetes is possible. Finally, progress continues in the field of islet transplantation, perhaps the ultimate frontier in diabetes management.

## Diabetes Treatment in the Pre-insulin Era

Diabetes is a chronic metabolic disease causing significant morbidity and premature cardiovascular mortality worldwide. According to the World Health Organization (WHO) approximately 537 million adults (ages 20–79 years) are living with diabetes today, and this number is predicted to rise to 643 million by 2030 and 783 million by 2045^1^.

The first description of a polyuric state resembling diabetes has been attributed to Hesy Ra, chief physician to the Egyptian Pharoah Djoser, nearly 5000 years ago^2^. The presence of sweetness in the urine was initially noted by the ancient Hindu physicians Charaka and Sushruta around 400–500 BC^3^. The term “diabetes” (from the Greek for siphon) has been attributed to Apollonius of Memphis in ancient Greece (around 250 BC), while another Greek physician, Aretaeus of Cappadocia (30–90 AD) described the condition as "the melting down of flesh and limbs into urine”^3^. John Rollo, a Scottish military surgeon is said to have first used the word “mellitus” (from the Latin for honey) in 1797^3^.

Although physicians in ancient times recognized the classic symptoms of diabetes and the sweetness of the urine, the treatments they used were empirical and included various herbs, chemicals, drugs and extreme diets to treat symptoms of the disease^3,4^.

In the mid-1600s, Thomas Willis introduced carbohydrate-restriction and limited his patients to a diet of milk and barley water boiled with bread^3,4^. In the 1700s, the “Meat Diet” was popularized by John Rollo^3,4^. The French physician Apollinaire Bouchardat (1809–1886), considered the “Father of Diabetology,” became the first to implement individualized therapy for patients, introducing exercise, and advocating daily urine testing “to keep track of the tolerance and to guard against a return of sugar without the patient’s knowledge^3,4^.” He also forbade milk because of its carbohydrate content, and “urged that patients eat as little as possible, and masticate carefully”^3,4^. In addition, he prescribed sodium bicarbonate, chalk, magnesia, citrates, tartrates, and ammonium and potassium salts. Around the end of the nineteenth century, Sir William Osler (1849–1919), the “Father of Modern American Medicine,” recommended that diabetes patients consume a diet of 65% fat, 32% protein, and 3% carbohydrate, and abstain from “all fruits and garden stuff”^3,4^. He further noted that “no one drug has directly curative influence” but that “opium alone stands the test of experience as a remedy capable of limiting the progress of the disease”^3^.

At the dawn of the twentieth century, Frederick Allen of The Rockefeller Institute^3^ introduced a diet that involved fasting for up to 10 days to clear glycosuria, followed by a restricted-calorie diet that provided mainly fat and protein (especially eggs) with the smallest amount of carbohydrates (mostly vegetables) necessary to sustain life. This regimen essentially starved people with severe diabetes to control the disease^3^. Elliot P. Joslin, the pioneer of diabetes care in the United States (US), embraced the Allen approach, but also used a treatment that began by withdrawing only fat^3–5^, and then protein after 2 days followed by a progressive lowering of carbohydrates in the diet to 10 g a day or until the patient’s urine was free of sugar! Sadly, despite all these heroic diets, the prognosis for patients remained uniformly grim until the discovery of insulin by Banting, Best and colleagues in Toronto, Canada, in 1921^6^.

## The Discovery of Insulin and the Age of Glucose Lowering

Insulin introduced a paradigm shift in diabetes management from extreme dietary carbohydrate restriction to effective lowering of blood glucose with insulin treatment. In subsequent years, several modifications were made to the insulin molecule to promote its stability and duration of action, included among these was NPH insulin (1946), still in use today. Finally, in 1955, the first oral anti-diabetic medication, the sulfonylurea (SU) carbutamide was introduced, followed by others in the same family including chlorpropamide, tolbutamide, glipizide, glyburide and glimiperide among others^7^. The first insulin sensitizer, the biguanide Metformin was introduced in Europe in 1957 and in the US in 1995. Notably, until the early 1990s, only three classes of drugs were available to treat diabetes – insulin, SUs and metformin. In the mid-1990s, several oral anti-diabetic agents were introduced including the alpha-glucosidase inhibitors (Acarbose, Miglitol and Voglibose), followed by the meglitinides (Nateglinide and Repaglinide)^7^, whose actions are similar to those of SUs, but of shorter duration. The late 1990s and early 2000s saw the introduction of the thiazolidinediones (TZDs), which are PPARγ agonists and potent insulin sensitizers^7^. However, issues related to liver toxicity led to the withdrawal of troglitazone from the market. Later concerns over CV safety with rosiglitazone led to its withdrawal^8^, and also to the FDA directive that all diabetes drugs demonstrate CV safety^9^. Pioglitazone is still available, with proven benefits for stroke prevention and diabetes prevention. However, the side effects of edema, fractures and heart failure (HF) precipitation limit its widespread use^7^. With the turn of the twenty-first century, the range of anti-hyperglycemic options broadened to include the first human insulin analogs followed by several other short- and long-acting analogs^7^, pramlintide (an injectable amylin analog) in 2005, and the dipeptidyl-peptidase inhibitors (DPP-4 inhibitors) which are oral agents in the incretin class of drugs in 2006. These medications (Sitagliptin Vildagliptin Saxagliptin Linagliptin, Alogliptin) inhibit DPP‑4 activity, increase endogenous incretin levels, and thereby promote glucose-dependent increases in insulin and inhibit glucagon secretion. Other drugs launching in the late 2000s include colesevelam (a bile acid sequestrant which activates liver farnesoid receptors) in 2008, and bromocriptine which activates hypothalamic dopamine receptors in 2009^7^.

Perhaps the most important classes of drugs to be introduced in the 2000s are the GLP-1 receptor agonists (GLP1-RA), the SGLT2-inhibitors (SGLT2i) and the dual GLP-1 receptor and GIP receptor agonists (GIP/GLP1-RA)^7^. The GLP1RA (Exenatide Liraglutide, Lixisenatide, Albiglutide, Dulaglutide and Semaglutide) directly act on the GLP-1 receptor to stimulate glucose-dependent insulin secretion and inhibit glucagon secretion. Their effects are far more potent than those of the DPP-4 inhibitors. In addition, they also reduce postprandial glucose excursions by slowing gastric motility and act centrally to increase satiety, leading to weight loss^7^. In 2022, the FDA approved the first combined GIP and GLP-1RA for the treatment of adults with type 2 diabetes (T2DM), with initial studies demonstrating superiority in both glycemic control and weight loss compared to GLP1-RA alone^10^. The SGLT2i drugs act through a novel mechanism to inhibit SGLT2 transporters in proximal renal tubules promoting glucosuria and lowering of blood glucose^7^. The robust cardio-renal benefits of these medications have transformed the landscape of diabetes treatment^11,12^, and led to these agents being recommended for cardio-renal risk reduction in high risk patients with T2DM^13^.

The primary purpose of all the above medications is to lower blood glucose, and they do so with variable efficacy. Insulin and the GLP-1RA and dual GLP-1RA/GIP-RA have very high efficacy; TZDs, SUs and metformin high efficacy; SGLT2i intermediate to high efficacy; and DPP-4i, AGI and colesevelam intermediate efficacy^13^. The improvements in glycemic control with medical therapy and lifestyle measures have clearly ameliorated polyuric symptoms and increased the life span of patients with diabetes. However, with increased life expectancy, patients are more prone to manifest the classic microvascular and macrovascular complications of diabetes^14^, broadening the goal of diabetes treatment to include prevention of these long-term complications.

## The Advent of the Cardiovascular Outcomes Trials and Cardio-renal Protection

In the 1990s, the DCCT and the UKPDS trials demonstrated the benefits of tight glucose control on the microvascular complications of diabetes such as retinopathy, nephropathy and neuropathy^15,16^. However, tight glucose control had marginal effects on macrovascular disease, and unexpectedly increased cardiovascular and all-cause mortality in the ACCORD study^17^. This was particularly concerning as the vast majority of patients with diabetes die from premature CV disease^18^. Concerns regarding the CV safety of anti-diabetic agents intensified when a meta-analysis suggested that the widely used rosiglitazone was associated with increased risk of myocardial infarction and CV death^8^. This prompted the FDA guidance in 2008, directing that any new diabetes therapy be evaluated for CV safety^9^. Following the FDA guidance, several large cardiovascular outcomes trials (CVOTs) were conducted and results with the GLP-1RA and SGLT2i have transformed the landscape of diabetes treatment^19–21^.

Initially, results from CVOTs with SGLT2i were neutral with regard to CV benefits. However, in 2015, the EMPA-REG OUTCOME trial demonstrated that empagliflozin, compared to placebo, significantly reduced the incidence of major adverse cardio-vascular events (MACE) comprising non-fatal MI, stroke and CV death in patients with T2DM and established CV disease^22^. These results, which occurred with optimal use of statins, blood pressure agents and RAAS blockers, surprised many in the diabetes community, and were felt to be chance findings. However, the CV benefits were replicated with other SGLT2i (canagliflozin and dapagliflozin)^19,21^. Simultaneously, CV benefits were also seen in the CVOTs conducted with the GLP-1RA, which have a significantly different mechanism of action. The first positive CV results with GLP-1RA were reported in the LEADER trial with liraglutide^23^ and have been subsequently confirmed as a class effect with albiglutide (HARMONY study) and dulaglutide (REWIND study). The SOUL study with semaglutide is ongoing and results are expected in 2024^25^.

The CVOTs also revealed that SGLT2i and GLP-1RA are associated with improved renal function. Although renal benefits of GLP1-RA have been observed only as secondary outcomes and in meta-analyses thus far^20^, the benefit from SGLT2i has been confirmed in large dedicated renal outcomes studies^24^. Thus, both SGLT2i and GLP1RA, in addition to effectively lowering blood glucose levels, also improve clinically significant cardiovascular and renal outcomes. In this context, it is important to note that there appear to be differences in the CV and renal benefits seen with SGLT2i and the GLP-1RA^26,27^. In the CVOTs to-date, the GLP-1RA have consistently demonstrated reduction in atherosclerotic cardiovascular disease (ASCVD) events in patients both with and without established ASCVD^26^, however, their effect on renal disease is confined to improvements in albuminuria without preventing progression to end stage kidney disease (ESRD—dialysis/kidney transplant) ESRD^27^. Further, the GLP-1RA do not have beneficial effects on HF in diabetes (REF). In contrast, the SGLT2i have modest benefits on atherosclerotic MACE confined to patients with established ASCVD^26,27^ but have robust benefits on reducing hospitalization for HF and progression of renal disease, regardless of existing ASCVD or a HF history. More importantly, unlike GLP-1RA, SGLT2i reduce hospitalization for HF and progression to ESRD in those with and without diabetes, as seen in the DAPA HF, DAPA CKD, CREDENCE and the EMPEROR PRESERVED/REDUCED studies^21,24^.

The above cardio-renal benefits have led to a major shift in international treatment guidelines. Both the American Diabetes Association (ADA) and the European Society for the Study of Diabetes (EASD) now recommend the use of SGLT2i and GLP-1RA as first-line treatment to reduce the risk of cardiorenal complications in individuals at high risk of CV disease, irrespective of metformin use and baseline/target glucose control^13^. The European Society of Cardiology (ESC) guidelines also recommend either a SGLT2i or a GLP-1RA as first-line treatment in people with T2DM at high CV risk, ahead of metformin^28^. However, it is important to note that in the CVOTs, most participants with diabetes were on at least one glucose-lowering medication (primarily metformin) at baseline^29^.

## The Case for Diabetes Prevention

The natural history of T2DM has been well defined, starting with a genetic predisposition and progression from normal glucose tolerance with insulin resistance to impaired glucose tolerance (pre-diabetes) and eventually to T2DM with β-cell failure^30^. The first large study to demonstrate that T2DM can be prevented was the Da Qing study from China published in 1995^31^. The authors randomized 577 men and women with impaired glucose tolerance (IGT) to the active intervention (*n* = 438) or control (*n* = 138). At 6 years, the cumulative incidence of diabetes was 67.7% in the control group compared with 43.8% in the diet group, 41.1% in the exercise group, and 46.0% in the diet-plus-exercise group (*P* < 0.05). After 30 years, compared with control, the combined intervention group still had a median delay in diabetes onset of 3.96 years (*p* = 0.0042) and had fewer microvascular complications, CV events, CV deaths, all-cause deaths and an average increase in life expectancy of 1.44 years^32^.

The next large study which evaluated diet and lifestyle in diabetes prevention was the Finnish Diabetes Prevention study (2001) which randomized 522 subjects with IGT (172 men and 350 women; mean age, 55 years; mean BMI 31 kg/m^2^) to either the intervention or the control group^33^. The intervention group received individualized counseling aimed at reducing weight and dietary fat intake, and increasing fiber intake and physical activity. The cumulative incidence of diabetes after four years was 11% in the intervention group and 23% in the control group, with a relative risk reduction of 58% (*p* < 0.001)^33^.

In contrast to the above studies which evaluated only diet and exercise, the US Diabetes Prevention Program (DPP, 2002) also evaluated the role of metformin in diabetes prevention^34^. In this study, 3234 pre-diabetic individuals (68% women, mean age 51 years, mean BMI 34 kg/m^2^) were randomized to placebo, metformin (850 mg twice daily), or a lifestyle-modification program targeting a minimum 7% weight loss and 150 min of physical activity per week. After an average follow-up of 2.8 years, as compared to placebo, lifestyle intervention reduced the incidence by 58% compared to only 31% with metformin. At 15 years, diabetes incidence in the DPP cohort was reduced by only 27% in the lifestyle intervention group and by 18% in the metformin group^35^. Nonetheless, these differences were still significant and the cumulative incidence of diabetes was 55%, 56% and 62% in the in the lifestyle, metformin and placebo groups, respectively. Interestingly, the prevalence of the aggregate microvascular outcomes at 15 years was not significantly different between the treatment groups in the total cohort (around 11–13%). However, in women (*n* = 1887) lifestyle intervention was associated with a significantly lower prevalence (8.7%) than in the placebo and metformin (~ 11%) groups. Notably, compared with participants who developed diabetes, those who did not develop diabetes had a 28% lower prevalence of microvascular complications (*p* < 0.0001), confirming the important role of hyperglycemia, per se, in the development of microvascular complications^35^.

The DPP study did not evaluate the effects of combining lifestyle plus metformin to prevent diabetes. This was done in the Indian Diabetes Prevention-1 (IDDP -1) study, in which 531 subjects with IGT (421 men, 110 women, mean age 45.9 years, BMI 25.8 kg/m^2^) were randomized into four groups^36^. Group 1 was the control, Group 2 was given advice on lifestyle modification (LSM), Group 3 was treated with metformin (MET) and Group 4 was given LSM plus MET. After 3 years, the cumulative incidences of diabetes were 55.0%, 39.3%, 40.5% and 39.5% in Groups 1–4, respectively. The relative risk reduction was 28.5% with LSM (*p* = 0.018), 26.4% with MET (*p* = 0.029) and 28.2% with LSM + MET (*p* = 0.022), as compared with the control group. Thus, in this study, although both lifestyle and metformin significantly reduced the incidence of diabetes in Asian Indians with IGT, surprisingly, there was no added benefit from combining them. The same group also studied whether combining the insulin sensitizer pioglitazone with lifestyle modification would enhance the efficacy of lifestyle modification in preventing T2DM in Asian Indians with IGT and found no additional effect of pioglitazone above that of placebo^37^. These findings are in distinction the robust effect on diabetes prevention seen with pioglitazone in the US ACTOS NOW study in which pioglitazone reduced the risk of conversion of IGT to T2DM by 72% despite significant weight gain^38^. In addition to the above studies, reduction in diabetes incidence has also been reported in dedicated prevention studies with troglitazone, rosiglitazone, acarbose and orlistat^39^. It is important to note that although medications like GLP1-RA, SGLT2i and GLP-1RA/GIPRA are associated with reductions in diabetes incidence, these benefits were seen in post-hoc analyses and not in dedicated prevention studies. Similarly bariatric surgery is also associated with substantial reductions is progression to diabetes^40^ and diabetes remission as discussed below.

## The Concept of Diabetes Remission

It is well known that intensive diet and lifestyle measures can lead to significant weight loss which may be sustained for long periods of time and lead to a regression from overt diabetes to normal glucose regulation in individuals with T2DM^41^. This usually occurs early in the course of disease and is associated with partial recovery of both insulin secretion and insulin sensitivity. Several terms have been used to describe this phenomenon, including resolution, reversal, remission, and cure of diabetes. In 2021, the ADA issued a Consensus Report on the “Definition and Interpretation of Remission in Type 2 Diabetes^41^,” which concluded that diabetes remission is the most appropriate term it strikes a balance between the fact that diabetes may not always be active while acknowledging that the improvement in glycemia may not be permanent and that frank diabetes may recur (^41^, Table 1). Notably, remission of diabetes can occur with diet/lifestyle measures, pharmacologic treatment and with bariatric surgery.

**Table 1:** Criteria for diabetes remission.

Remission should be defined as a return of *HbA*_*1c*_ *to* < *6.5% (*< *48 mmol/mol)* that occurs spontaneously or following an intervention and that persists for *at least 3 months* in the absence of usual glucose-lowering pharmacotherapy
When HbA_1c_ is determined to be an unreliable marker of chronic glycemic control, *FPG* < *126 mg/dL (*< *7.0 mmol/L)* or *eA1C* < *6.5%* calculated from CGM values can be used as alternate criteria
Testing of HbA_1c_ to document a remission should be performed just prior to an intervention and no sooner than 3 months after initiation of the intervention and withdrawal of any glucose-lowering pharmacotherapy
Subsequent testing to determine long-term maintenance of a remission should be done at least yearly thereafter, together with the testing routinely recommended for potential complications of diabetes
Remission should be defined as a return of *HbA*_1c_ to < *6.5% (*< *48 mmol/mol)* that occurs spontaneously or following an intervention and that persists for *at least 3 months* in the absence of usual glucose-lowering pharmacotherapy
When HbA_1c_ is determined to be an unreliable marker of chronic glycemic control, *FPG* < *126 mg/dL (*< *7.0 mmol/L)* or *eA1C* < *6.5%* calculated from CGM values can be used as alternate criteria
Testing of HbA_1c_ to document a remission should be performed just prior to an intervention and no sooner than 3 months after initiation of the intervention and withdrawal of any glucose-lowering pharmacotherapy
Subsequent testing to determine long-term maintenance of a remission should be done at least yearly thereafter, together with the testing routinely recommended for potential complications of diabetes

### Intensive Weight Management and Diabetes Remission

The DiRECT study in the UK assessed whether intensive weight management within routine primary care increased remission of T2DM in patients diagnosed within the past six years and not on insulin^42^. In an open-label, cluster-randomized trial, 306 individuals (20–65 years) were randomized to an intervention group (*n* = 157) that underwent total diet replacement (825–853 kcal/day formula diet for 3–5 months), progressive food reintroduction (2–8 weeks), and structured support for long-term weight loss maintenance, or a control group (*n* = 149). Mean bodyweight by 10.0 kg versus 1.0 kg and diabetes remission was achieved in 46% vs 4% of participants in the intervention versus control group, respectively. Notably, at 12 months, almost half of the participants achieved remission to a non-diabetic state off antidiabetic drugs and at 24 months, remission persisted in more than a third of people with T2DM and was linked to the extent of sustained weight loss^43^.

***Bariatric Surgery and Diabetes Remission:*** compared to intensive diet/lifestyle, more robust rates of diabetes remission are achieved after bariatric surgery, with Roux-en-Y gastric bypass (RYGB) being associated with greater remission rates than sleeve gastrectomy^40^. In a large retrospective, observational study of 5928 patients with T2DM at the time of surgery, over an average follow-up of nearly 6 years, 71% of patients experienced remission of T2DM (mean time to remission 1.0 year), with weight loss after bariatric surgery being strongly associated with initial T2DM remission up to a threshold of 20% total weight loss.

***Pharmacologic Treatment and Diabetes Remission:*** whether a remission can be diagnosed in the setting of ongoing pharmacotherapy is a complex question. In many patients with short duration of diabetes, glycemic control can be achieved by short-term use of glucose-lowering drugs, especially the GLP-1RA and the recently approved dual GIP1 and GLP-1RA which are associated with robust reductions in body weight. In the SURPASS-1 trial 705 individuals with short duration T2DM (mean 4.7 years, mean HbA1c ~ 8.0%) were randomized to placebo or escalating doses of tirzepatide a novel “twincretin” with glucagon-like peptide 1 and glucose-dependent insulinotropic polypeptide receptor agonist activity, recently approved by the FDA for the treatment of type 2 diabetes^44^. After 40 weeks of treatment, the mean HbA1c decreased from baseline by ~ 2.0% in the tirzepatide group, along with weight loss of 7 to 9.5 kg from a baseline of 5.5 kg. Notably, more participants on tirzepatide than on placebo met HbA1c targets of < 7·0% (~ 90% vs 20%) and 6·5% or less (81–86% vs 10%) and 31–52% of patients on tirzepatide versus 1% on placebo reached an HbA1c of less than 5·7%, which is in the normal range. Whether these patients will continue to remain in the normal range after withdrawing tirzepatide treatment is not known. Indeed, it has been argued that withdrawing medications may not be ideal since these glucose-lowering medications also have positive effects on protecting the heart and kidneys, thus stopping these medications may not be the best course of action to limit the progression of complications long-term.

## The Role of Technology in Diabetes Management

### Advances in Glucose Monitoring and Insulin Delivery

In the past decade, rapid advances in technology have led to the introduction of sophisticated continuous glucose monitoring systems (CGMS) which not only provide near-real-time glucose readings, but also communicate with state-of-the-art insulin pumps which correlate insulin delivery with glucose trends. Hybrid closed-loop systems partially automate insulin dosing, requiring only manual mealtime boluses and occasional correction boluses. This has led to significant improvements in glucose control and reductions in hypoglycemia^45^. In registry and case–control longitudinal data, pump use has been associated with fewer CV events and reduction of CV disease and all-cause mortality^46^. Studies on bi-hormonal (insulin and glucagon) systems are also ongoing. Of note, insulin pumps and CGMS are expensive and primarily used in patients with type 1 diabetes mellitus (T1DM). With ongoing improvements in scanning technology and tubeless insulin pods, the cost of CGMS and insulin pumps has decreased and may become available at a reasonable cost for patients with T2DM as well.

The goal in this field is to develop long term, implantable glucose sensors and fully automated insulin delivery systems which use artificial intelligence to seamlessly maintain glucose in the normal range without the need for human intervention^47^. Together with diabetes technology and telemedicine, the use of artificial intelligence (AI) is slowly coming of age and may soon present a paradigm shift in diabetic management through data-driven precision care. AI supports the development of predictive models that can be implemented at the individual level to refine glycemic control, or at population level to estimate the risk of diabetes and its related complications across diverse patient groups^48^.

### The Advent of Telemedicine

Telemedicine can be useful for the management of diabetes mellitus and can also be cost-effective^49^. Remote monitoring of glucose levels improves A1C levels in people with poor glucose control. When multiple daily injections of insulin are required, continuous glucose monitoring improves glycemic control and increases patient satisfaction^49^. Until recently, the use of telemedicine has been minimal. However, social-distancing requirements during the COVID-19 pandemic, led to a rapid acceleration of telemedicine, allowing providers to interact with patients virtually and safely^50^. Telemedicine increases convenience for patients and providers alike and eliminates the risk of spreading infections in the clinic. However, it must be remembered that although telemedicine can be used to deliver effective diabetes care and complement current diabetes management strategies, it cannot replace all in-person consultations. Patients with complex health needs, and those who require a physical examination are not suitable for telemedicine.

### The Role of Precision Medicine

Precision medicine is an emerging approach for disease prevention and treatment that considers how individual variability in genes, environment, and lifestyle impact disease^51^. The precision medicine approach allows doctors and researchers to predict more accurately which treatment and prevention strategies for a particular disease will work in which groups of people^52^. This is in contrast to a one-size-fits-all approach, in which disease treatment and prevention strategies are developed with less consideration for the differences between individuals. Precision medicine has the potential to offer direct clinical benefits to patients and to be more cost-efficient for society as time and resources are not wasted on less efficacious treatments. Compared with oncology, the role of precision medicine in diabetes management is less clear given the heterogeneous nature of T2DM, and the fact that diabetes medications are usually selected based on comorbidities, cost and side effects, rather than on the specific pathophysiology underlying disease in the individual patient.

## The Last Frontier in Diabetes Management

### Islet Transplantation

**Figure 1:**
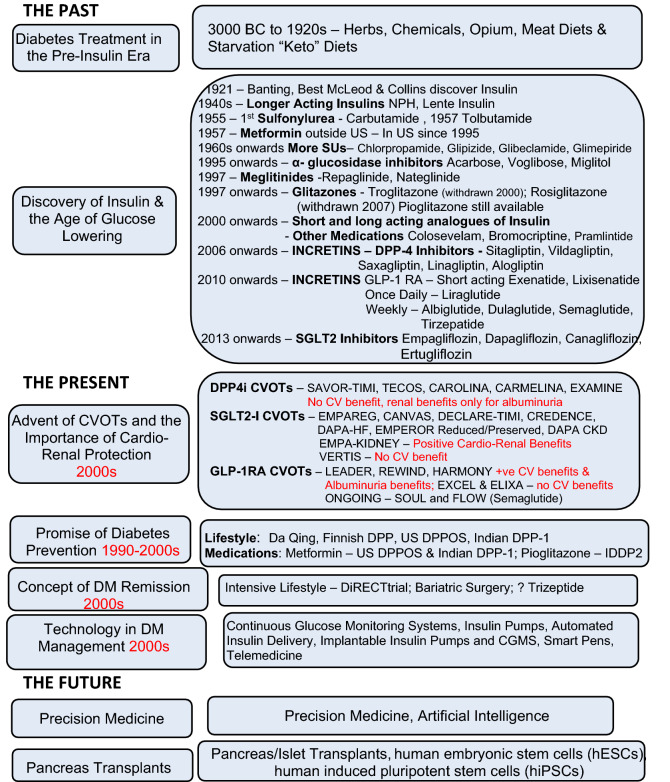
Landmarks in diabetes management over the ages.

Loss of β-cell function is an important feature of diabetes. In T1DM autoimmune-mediated β-cell destruction leads to absolute insulin deficiency, while T2DM is characterized by relative insulin deficiency due to β-cell dysfunction, often in the setting of insulin resistance. Daily insulin injection treatment is the standard care for patients with T1DM and those with late-stages of T2DM. Currently, the use of automated insulin delivery with insulin pumps and CGMS has simplified insulin delivery and reduced the occurrence of hypoglycemia^45^. However, subcutaneous insulin delivery differs from endogenous insulin secretion which occurs directly into the hepatic portal system without producing peripheral hyperinsulinemia. A promising avenue is β-cell replacement through whole pancreas or islet cell transplantation^53,54^. This approach not only restores physiologic insulin secretion but also reduces hypoglycemia risk by partially restoring glucagon secretion. Both pancreas and islet transplantation require lifelong immunosuppression to prevent graft rejection. Data from pancreas and islet transplantation registries show a higher rate of insulin independence with pancreas transplantation compared with islet transplantation (85% versus 50% at one year), but also increased morbidity due to the need for open surgery^55^. Although rates of long-term insulin independence are lower, islet transplantation is less invasive and may allow for successive procedures. Unfortunately, donor shortage hinders the widespread implementation of these therapies. However, advances in stem cell technology may be able to bridge this gap in the future. Researchers have successfully differentiated human induced pluripotent stem cells and embryonic stem cells into β-like cells that are able to secrete insulin in response to variable glucose levels [55]. Though still preliminary, these studies are promising, and may represent the future of diabetes management (Fig. 1).

## Conclusion

We have come a long way in the management of diabetes—from herbs, chemicals and extreme starvation diets before the advent of insulin to potent oral agents and injectables which improve glycemia, and in the case of the SGLT2i and the GLP-1RA, provide robust cardio-renal protection as well. The dual GIP1/GLP1RA tirzepatide even induces remission of diabetes along with substantial weight loss similar to that seen with bariatric surgery procedures. Remarkably, despite the passage of a century, insulin and its various analogues still remain a cornerstone of diabetes treatment in those with T1DM and in patients with long-standing T2DM. Like insulin, metformin has also stood the test of time for over six decades, while the SUs have slowly lost favor in the last two decades due to side effects of hypoglycemia and weight gain. Similarly, the thiazolidinediones, despite being potent insulin sensitizers and conferring stroke benefits, are not widely used due to issues with weight gain and edema. However, both SUs and TZDs are still widely used when cost is an issue. Lately, several advances have been made in diabetes technology with the advent of smart insulin pens, CGMS and insulin pumps which have revolutionized insulin treatment, especially in patients with T1DM. The recent COVID-19 epidemic has given rise to the rapid ascent of telemedicine. Artificial Intelligence has the potential to introduce a paradigm shift in diabetes management through data-driven precision care. Sadly, many technologies and newer agents remain out of reach to the vast majority of patients with diabetes. Health authorities and agencies across the world will need to work together to address issues with equity and access to affordable diabetes care. There is hope that with the introduction of generic, and potentially less expensive SGLT2i and GLP-1RA, there will be more widespread use of these agents, along with more education of healthcare providers on the risks/benefits of these medications which provide robust cardio-renal protection. Progress also continues in the field of islet transplantation, perhaps the final frontier in diabetes management. Finally, let us not forget that diet and lifestyle measures still remain the foundation of management and can help prevent the progression from pre-diabetes to diabetes. As the old adage goes: “An ounce of prevention is worth a pound of cure.”

## Data Availability

https://grammarist.com/proverb/an-ounce-of-prevention-is-worth-a-pound-of-cure/

## References

[1] https://diabetesatlas.org/

[2] Loriaux DL (2006). Diabetes and the Ebers Papyrus: 1552 BC. The Endocrinologist.

[3] Zinman B, Skyler JS, Riddle MC, Ferrannini E (2017). Diabetes research and care through the ages. Diabetes Care.

[4] Frederick M. Allen, Edgar Stillman, and Reginald Fitz, M.D. Total Dietary Regulation in the Treatment of Diabetes. Monographs of the Rockefeller Institute for Medical Research, No. 11, Oct. 15,1919. The Rockefeller Institute for Medical Research, New York.

[5] Joslin EP. Treatment of diabetes mellitus. 2nd ed. Philadelphia, Lea & Febiger, 1917, p. 409

[6] Rydén L, Lindsten J (2021). The history of the Nobel prize for the discovery of insulin. Diabetes Res Clin Pract.

[7] Tahrani AA, Barnett AH, Bailey CJ (2016). Pharmacology and therapeutic implications of current drugs for type 2 diabetes mellitus. Nat Rev Endocrinol.

[8] Nissen SE, Wolski K (2007). Effect of rosiglitazone on the risk of myocardial infarction and death from cardiovascular causes. N Engl J Med.

[9] https://www.fda.gov/media/135936/download

[10] Frías JP, Davies MJ, Rosenstock J, Pérez Manghi FC, Fernández Landó L, Bergman BK, Liu B, Cui X, Brown K; SURPASS-2 Investigators. Tirzepatide versus Semaglutide Once Weekly in Patients with Type 2 Diabetes. N Engl J Med. 2021;385(6):503–515. 10.1056/NEJMoa2107519 34170647

[11] Zelniker TA, Wiviott SD, Raz I, Im K (2019). SGLT2 inhibitors for primary and secondary prevention of cardiovascular and renal outcomes in type 2 diabetes: a systematic review and meta-analysis of cardiovascular outcome trials. Lancet.

[12] Brown E, Heerspink HJL, Cuthbertson DJ, Wilding JPH (2021). SGLT2 inhibitors and GLP-1 receptor agonists: established and emerging indications. Lancet.

[13] Davies MJ, Aroda VR, Collins BS, Gabbay RA, Green J, Maruthur NM, Rosas SE, Del Prato S, Mathieu C, Mingrone G, Rossing P, Tankova T, Tsapas A, Buse JB. Management of Hyperglycemia in Type 2 Diabetes, 2022. A Consensus Report by the American Diabetes Association (ADA) and the European Association for the Study of Diabetes (EASD). Diabetes Care. 2022 Sep 23:dci220034. doi: 10.2337/dci22-0034. Epub ahead of print. 10.2337/dci22-0034 PMC10008140 36148880

[14] Forbes JM, Cooper ME (2013). Mechanisms of diabetic complications. Physiol Rev.

[15] Diabetes Control and Complications Trial Research Group, Nathan DM, Genuth S, Lachin J, et al. The effect of intensive treatment of diabetes on the development and progression of long-term complications in insulin-dependent diabetes mellitus. N Engl J Med. 1993 Sep 30;329(14):977–86. 10.1056/NEJM199309303291401 8366922

[16] Intensive blood-glucose control with sulphonylureas or insulin compared with conventional treatment and risk of complications in patients with type 2 diabetes (UKPDS 33). UK Prospective Diabetes Study (UKPDS) Group. Lancet. 1998 Sep 12;352(9131):837–53. 9742976

[17] Action to Control Cardiovascular Risk in Diabetes Study Group, Gerstein HC, Miller ME, et al. Effects of intensive glucose lowering in type 2 diabetes. N Engl J Med. 2008;358(24):2545–59. 10.1056/NEJMoa0802743 PMC4551392 18539917

[18] Fisher M, Shaw S (2001). Diabetes – a state of premature cardiovascular death. Pract Diabetes Int.

[19] Lin DS, Lee JK, Hung CS, Chen WJ (2021). The efficacy and safety of novel classes of glucose-lowering drugs for cardiovascular outcomes: a network meta-analysis of randomised clinical trials. Diabetologia.

[20] Sattar N, Lee MMY, Kristensen SL, Branch KRH, Del Prato S, Khurmi NS, Lam CSP, Lopes RD, McMurray JJV, Pratley RE, Rosenstock J, Gerstein HC (2021). Cardiovascular, mortality, and kidney outcomes with GLP-1 receptor agonists in patients with type 2 diabetes: a systematic review and meta-analysis of randomised trials. Lancet Diabetes Endocrinol.

[21] Htoo PT, Buse J, Cavender M, Wang T, Pate V, Edwards J, Stürmer T (2022). Cardiovascular effectiveness of sodium-glucose cotransporter 2 inhibitors and glucagon-like peptide-1 receptor agonists in older patients in routine clinical care with or without history of atherosclerotic cardiovascular diseases or heart failure. J Am Heart Assoc.

[22] Zinman B, Wanner C, Lachin JM, EMPA-REG OUTCOME Investigators. Empagliflozin, Cardiovascular Outcomes, and Mortality in Type 2 Diabetes. N Engl J Med. 2015;373(22):2117–28. 10.1056/NEJMoa1504720 26378978

[23] Marso SP, Daniels GH, Brown-Frandsen K,; LEADER Steering Committee; LEADER Trial Investigators. Liraglutide and Cardiovascular Outcomes in Type 2 Diabetes. N Engl J Med. 2016;375(4):311–22. 10.1056/NEJMoa1603827 PMC4985288 27295427

[24] Nuffield Department of Population Health Renal Studies Group; SGLT2 inhibitor Meta-Analysis Cardio-Renal Trialists' Consortium. Impact of diabetes on the effects of sodium glucose co-transporter-2 inhibitors on kidney outcomes: collaborative meta-analysis of large placebo-controlled trials. Lancet. 2022;400(10365):1788–1801. 10.1016/S0140-6736(22)02074-8 PMC7613836 36351458

[25] https://clinicaltrials.gov/ct2/show/NCT03819153

[26] Hupfeld C, Mudaliar S (2019). Navigating the "MACE" in cardiovascular outcomes Trials and decoding the relevance of atherosclerotic cardiovascular disease benefits versus heart failure benefits. Diabetes Obes Metab.

[27] Ekanayake P, Mudaliar S (2021). Changing the diabetes treatment paradigm from glucose control to cardiorenal protection. Indian J Med Res.

[28] Visseren FLJ, Mach F, Smulders YM; ESC Scientific Document Group. 2021 ESC Guidelines on cardiovascular disease prevention in clinical practice. Eur J Prev Cardiol. 2021 Sep 24:zwab154. 10.1093/eurjpc/zwab154 34558602

[29] Zaccardi F, Khunti K, Marx N, Davies MJ (2020). First-line treatment for type 2 diabetes: is it too early to abandon metformin?. Lancet.

[30] William C Knowler, K M V Narayan, Robert L Hanson, Robert G Nelson, Peter H Bennett, Jaakko Tuomilehto, Bengt Scherstén, David J Pettitt; Preventing Non-Insulin-Dependent Diabetes. Diabetes 1 May 1995; 44 (5): 483–488. 10.2337/diab.44.5.483 7729603

[31] Pan XR, Li GW, Hu YH (1997). Effects of diet and exercise in preventing NIDDM in people with impaired glucose tolerance: the Da Qing IGT and Diabetes Study. Diabetes Care.

[32] Gong Q, Zhang P, Wang J, Ma J, An Y, Chen Y, Zhang B, Feng X, Li H, Chen X, Cheng YJ, Gregg EW, Hu Y, Bennett PH, Li G; Da Qing Diabetes Prevention Study Group. Morbidity and mortality after lifestyle intervention for people with impaired glucose tolerance: 30-year results of the Da Qing Diabetes Prevention Outcome Study. Lancet Diabetes Endocrinol. 2019;7(6):452–461. 10.1016/S2213-8587(19)30093-2. 10.1016/S2213-8587(19)30093-2 PMC8172050 31036503

[33] Tuomilehto J, Lindstrom J, Eriksson JG (2001). Prevention of type 2 diabetes mellitus by changes in lifestyle among subjects with impaired glucose tolerance. N Engl J Med.

[34] Knowler WC, Barrett-Connor E, Fowler SE, Hamman RF, Lachin JM, Walker EA, Nathan DM; Diabetes Prevention Program Research Group. Reduction in the incidence of type 2 diabetes with lifestyle intervention or metformin. N Engl J Med. 2002;346(6):393–403. 10.1056/NEJMoa012512 PMC1370926 11832527

[35] Diabetes Prevention Program Research Group (2015). Long-term effects of lifestyle intervention or metformin on diabetes development and microvascular complications over 15-year follow-up: the Diabetes Prevention Program Outcomes Study. Lancet Diabetes Endocrinol.

[36] Ramachandran A, Snehalatha C, Mary S, Mukesh B, Bhaskar AD, Vijay V; Indian Diabetes Prevention Programme (IDPP). The Indian Diabetes Prevention Programme shows that lifestyle modification and metformin prevent type 2 diabetes in Asian Indian subjects with impaired glucose tolerance (IDPP-1). Diabetologia. 2006;49(2):289–97. 10.1007/s00125-005-0097-z 16391903

[37] Ramachandran A, Snehalatha C, Mary S, Selvam S, Kumar CK, Seeli AC, Shetty AS (2009). Pioglitazone does not enhance the effectiveness of lifestyle modification in preventing conversion of impaired glucose tolerance to diabetes in Asian Indians: results of the Indian Diabetes Prevention Programme-2 (IDPP-2). Diabetologia.

[38] DeFronzo RA, Tripathy D, Schwenke DC, Banerji M, Bray GA, Buchanan TA, Clement SC, Henry RR, Hodis HN, Kitabchi AE, Mack WJ, Mudaliar S, Ratner RE, Williams K, Stentz FB, Musi N, Reaven PD; ACT NOW Study. Pioglitazone for diabetes prevention in impaired glucose tolerance. N Engl J Med. 2011;364(12):1104–15. 10.1056/NEJMoa1010949 21428766

[39] Knowler WC, Crandall JP (2019). Pharmacologic randomized clinical trials in prevention of type 2 diabetes. Curr Diab Rep.

[40] Barthold D, Brouwer E, Barton LJ, Arterburn DE, Basu A, Courcoulas A, Crawford CL, Fedorka PN, Fischer H, Kim BB, Mun EC, Murali SB, Reynolds K, Yoon TK, Zane RE, Coleman KJ (2022). Minimum threshold of bariatric surgical weight loss for initial diabetes remission. Diabetes Care.

[41] Riddle MC, Cefalu WT, Evans PH, Gerstein HC, Nauck MA, Oh WK, Rothberg AE, le Roux CW, Rubino F, Schauer P, Taylor R, Twenefour D (2021). Consensus report: definition and interpretation of remission in type 2 diabetes. Diabetes Care.

[42] Lean ME, Leslie WS, Barnes AC (2018). Primary care-led weight management for remission of type 2 diabetes (DiRECT): an open-label, cluster-randomised trial. Lancet.

[43] Lean MEJ, Leslie WS, Barnes AC (2019). Durability of a primary care-led weight-management intervention for remission of type 2 diabetes: 2-year results of the DiRECT open-label, cluster-randomised trial. Lancet Diabetes Endocrinol.

[44] Rosenstock J, Wysham C, Frías JP, Kaneko S, Lee CJ, Fernández Landó L, Mao H, Cui X, Karanikas CA, Thieu VT (2021). Efficacy and safety of a novel dual GIP and GLP-1 receptor agonist tirzepatide in patients with type 2 diabetes (SURPASS-1): a double-blind, randomised, phase 3 trial. Lancet.

[45] Sherwood JS, Russell SJ, Putman MS (2020). New and emerging technologies in type 1 diabetes. Endocrinol Metab Clin North Am.

[46] Pauley ME, Tommerdahl KL, Snell-Bergeon JK, Forlenza GP. Continuous Glucose Monitor, Insulin Pump, and Automated Insulin Delivery Therapies for Type 1 Diabetes: An Update on Potential for Cardiovascular Benefits. Curr Cardiol Rep. 2022. 10.1007/s11886-022-01799-x. Epub ahead of print. 10.1007/s11886-022-01799-x PMC9589770 36279036

[47] Dassau E, Renard E, Place J, Farret A, Pelletier MJ, Lee J, Huyett LM, Chakrabarty A, Doyle FJ, Zisser HC (2017). Intraperitoneal insulin delivery provides superior glycaemic regulation to subcutaneous insulin delivery in model predictive control-based fully-automated artificial pancreas in patients with type 1 diabetes: a pilot study. Diabetes Obes Metab.

[48] Ellahham S (2020). Artificial intelligence: the future for diabetes care. Am J Med.

[49] Mullur RS, Hsiao JS, Mueller K (2022). Telemedicine in diabetes care. Am Fam Physician.

[50] Betancourt JA, Rosenberg MA, Zevallos A, Brown JR, Mileski M (2020). The impact of COVID-19 on telemedicine utilization across multiple service lines in the United States. Healthcare (Basel).

[51] Griffin S (2022). Diabetes precision medicine: plenty of potential, pitfalls and perils but not yet ready for prime time. Diabetologia.

[52] Collins FS, Varmus H (2015). A new initiative on precision medicine. N Engl J Med.

[53] Bourgeois S, Sawatani T, Van Mulders A, De Leu N, Heremans Y, Heimberg H, Cnop M, Staels W (2021). Towards a functional cure for diabetes using stem cell-derived beta cells: are we there yet?. Cells.

[54] Cayabyab F, Nih LR, Yoshihara E (2021). Advances in pancreatic islet transplantation sites for the treatment of diabetes. Front Endocrinol (Lausanne).

[55] Maffi P, Scavini M, Socci C, Piemonti L, Caldara R, Gremizzi C, Melzi R, Nano R, Orsenigo E, Venturini M, Staudacher C, Del Maschio A, Secchi A. Risks and benefits of transplantation in the cure of type 1 diabetes: whole pancreas versus islet transplantation. A single center study. Rev Diabet Stud. 2011 Spring;8(1):44–50. 10.1900/RDS.2011.8.44 PMC3143676 21720672

[56] https://www.uptodate.com/contents/pancreas-and-islet-transplantation-in-diabetes-mellitus

